# Association of ultra-processed foods with phenotypic age acceleration in US adults: a mediation analysis of body mass index in the NHANES

**DOI:** 10.3389/fnut.2025.1485456

**Published:** 2025-03-24

**Authors:** Weiliang Kong, Yilian Xie, Mengyuan Cen, Kunlong Xiong

**Affiliations:** ^1^Department of Respiratory and Critical Care Medicine, The First Affiliated Hospital of Ningbo University, Ningbo University, Ningbo, China; ^2^Department of Hepatology, The First Affiliated Hospital of Ningbo University, Ningbo University, Ningbo, China

**Keywords:** ultra-processed foods, phenotypic age acceleration, body mass index, NHANES, biological aging, dietary pattern

## Abstract

**Background:**

The rising intake of ultra-processed foods (UPFs) has been linked to adverse health outcomes, yet its impact on aging acceleration remains unclear.

**Objective:**

This study aimed to examine the association between the percentage of total daily calories (%Kcal) and grams (%Gram) from UPFs and phenotypic age acceleration (PhenoAgeAccel).

**Methods:**

Data from 12,079 adults in the NHANES 2005–2010 cycles were analyzed. The relationship between UPFs intake and PhenoAgeAccel was assessed using multivariable linear regression and restricted cubic splines, with adjustments for relevant covariates. The mediating role of body mass index (BMI) was also explored.

**Results:**

A significant positive linear association was observed between UPFs intake (%Gram) and PhenoAgeAccel, with the highest quartile showing an increase of 0.60 (95% CI: 0.15, 1.05; *p* for trend = 0.039), but no association was found between UPFs intake (%Kcal) and PhenoAgeAccel. Mediation analysis indicated that BMI mediated 27.5% of the association between UPFs intake (%Gram) and PhenoAgeAccel. Sensitivity analyses confirmed the robustness of the results.

**Conclusion:**

Higher intake of UPFs intake (%Gram) is positively associated with PhenoAgeAccel, with BMI playing a significant mediating role.

## Introduction

Aging is a complex physiological process driven by multiple biological mechanisms. These mechanisms involve various dimensions of cells, tissue, and organs, and are closely linked to nutritional, environmental, psychosocial, and demographic factor. The aging process also leads to various adverse health outcomes, which are strongly associated with the onset and progression of chronic diseases such as cardiovascular disease (CVD), cancer, osteoporosis, and neurodegenerative disorders (1). Moreover, the presence of these diseases can, in turn, accelerate the aging process. Therefore, aging is not only a manifestation of natural physiological processes but also the result of complex pathological interactions (1). In recent years, global trends in population aging have become increasingly pronounced. According to the 2023 World Population Prospects, the proportion of individuals aged 60 years and above currently stands at 12.3%, and this figure is projected to rise to 22% by 2050 (2). Population aging exacerbates the global burden of chronic diseases, imposing significant social and economic pressures. As a result, the search for effective interventions to mitigate aging has become a critical area of focus in global public health. Today, researchers have developed various methods to measure biological aging, each with notable differences in focus and techniques. Currently, the main approaches to assessing biological aging include phenotypic age (3), biological age (4), leukocyte telomere length (5), and metabolic age scores (6), etc. Each methods reflects distinct aspects of the aging process and holds specific value in aging research.

Dietary factors play a crucial role in influencing the aging process (7, 8). Previous studies have indicated that various nutrients, such as dietary fiber, high-quality carbohydrates, plant proteins, and omega-3 polyunsaturated fatty acids (PUFAs) (9), along with certain dietary patterns rich in antioxidants, anti-inflammatory agents, and overall healthy food choices (10, 11), may slow down the aging process through mechanisms that reduce inflammation and oxidative stress. However, despite the benefits of healthy dietary patterns, with the development of modern society and the economy, the consumption of ultra-processed foods (UPFs) has increased rapidly worldwide (12). UPFs refer to highly industrialized processed foods that typically contain large amounts of sugars, fats, and industrial additives, making them significantly different from the components and processing methods found in traditional diets (13). High intake of UPFs has been shown to be closely associated with adverse health outcomes such as obesity, type 2 diabetes, and cardiovascular diseases (14). Given the potential negative impacts of UPFs on health and the limited research exploring the associations between UPFs intake and aging, this study aims to address this research gap by examining the association between UPFs intake and phenotypic age acceleration (PhenoAgeAccel) in a nationally representative sample of U.S. adults.

## Methods

### Design

**Figure 1:**
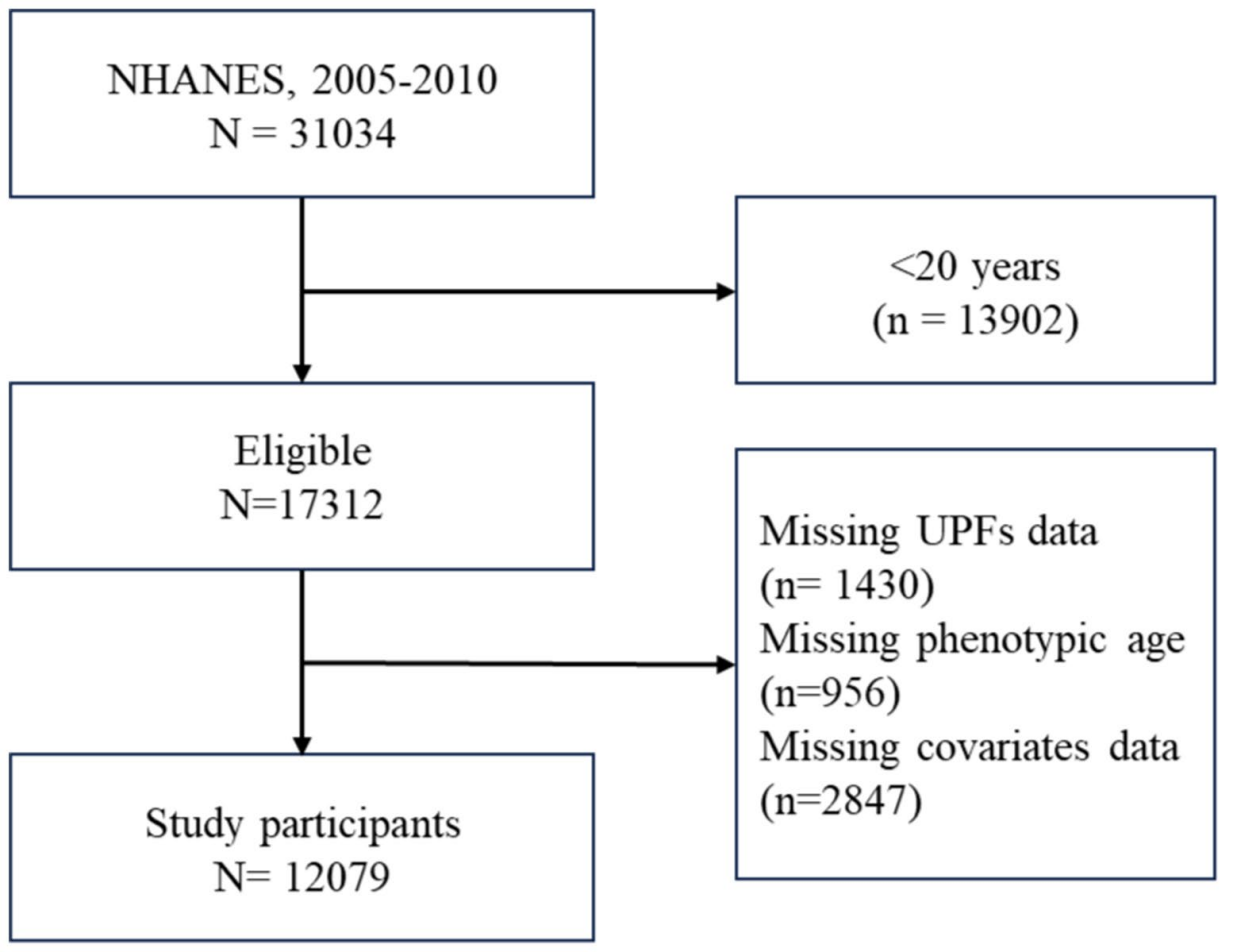
Participants flow chart.

This cross-sectional study drew upon data publicly available from the National Health and Nutrition Examination Survey (NHANES) to analyze the relationship between UPFs consumption and PhenoAgeAccel. Adults who responded to relevant questions concerning demographics, socioeconomic factors, dietary intake, chronic diseases, phenotypic age from 2005 to 2010 cycles were included in the analysis. After applying additional inclusion and exclusion criteria, participants were excluded if they were under 20 years old, lacked UPFs dietary data, phenotypic age composition data, or had missing covariate data. As a result, 12,079 participants remained available for the study. Detailed information is represented in Figure 1.

### UPFs

Trained interviewers used the USDA Automated Multiple-Pass Method to collect in-person 24-h dietary recalls. The NOVA food classification system was applied to the Food and Nutrient Database for Dietary Studies (FNDDS) to identify all food items classified as UPFs. Food composition was determined using the 8-digit food code, with all consumed food and beverages recorded in grams and then converted into calories using the FNDDS.

Then we calculated the percentage of total daily calories (%Kcal) and grams (%Gram) intake from UPFs based on the two-day average as indicators of UPFs intake. These indicators were also divided into quartiles to serve as an exposure variable. A more detailed description of the NOVA classification system can be found in our previous reports (15). The reliability and validity of classifying UPFs categories in 24-h dietary recall data by NOVA have been well-established (13).

### PhenoAgeAccel

Biological aging is measured by phenotypic age, which utilize different biomarkers and calculation methods.

Phenotypic Age was calculated using the following formula (3).


$$\mathrm{Phenotypic}\;\mathrm{Age}=141.50+\frac{\ln\left[-0.00553\times \ln\left(1-\mathrm{MortalityScore}\right)\right]}{0.09165}$$

where:


$$\mathrm{MortalityScore}=1-\exp\left(\frac{-1.51714\times \exp\left(xb\right)}{0.0076927}\right)$$

and:


$$\begin{array}{l} xb=-19.907-0.0336\times \mathrm{Albumin}+0.0095\times \\ \mathrm{Creatinine}+0.1953\times \mathrm{Glucose}+\\ 0.0954\times \mathrm{LnCRP}-0.0120\times \\ \mathrm{Lymphocyte}\;\mathrm{Percentage}+\\ 0.0268\times \mathrm{Mean}\;\mathrm{Cell}\;\mathrm{Volume}+\\ 0.3306\times \mathrm{Erythrocyte}\;\\ \mathrm{Distribution}\;\mathrm{Width}+0.0019\times \\ \mathrm{Alkaline}\;\mathrm{Phosphatase}+\\ 0.0554\times \mathrm{Leukocyte}\;\mathrm{Count}+\\ 0.0804\times \mathrm{chronological}\;\mathrm{age} \end{array}$$

To adjust for the effects of chronological age, we defined PhenoAgeAccel by calculating the residuals from a regression of phenotypic age on chronological age.

We also classify participants into accelerated and delayed aging group with PhenoAgeAccel ≥0 and PhenoAgeAccel <0, respectively.

### Covariation

Our analysis encompassed a range of covariates previously demonstrated or assumed to be associated with UPFs intake and biological aging. These covariates included age, as continuous variables or categorized as <40, 40–59, and ≥ 60 years; sex (female, male); racial/ethnicity, reclassified as Non-Hispanic Black, Non-Hispanic White, Mexican American, and others; poverty-to-income ratio (PIR), categorized as low (<1.3), middle (1.3–3.5), and high (>3.5); BMI according to World Health Organization (WHO) classifications: normal, overweight, and obese; education level (reclassified as college or higher, middle school or lower, and high school); alcohol consumption (categorized as never, former, mild, moderate, and heavy) (16, 17); smoking status (current, former, never); physical activity level (active, inactive, moderate, others (18)), presence of hypertension, diabetes mellitus (DM), cardiovascular disease(CVD). Hypertension and DM were determined through index measurements, medication usage, and self-reports, while CVD was self-reported. Additionally, we considered a healthy dietary score calculated using the Healthy Eating Index-2015 (HEI-2015), as well as energy and protein intake, derived from the mean of two days’ daily energy and protein intake.

### Statistical methods

Due to the complexity of NHANES sampling design, dietary day one sample weight was used for analysis. For baseline characteristics, continuous variables were expressed as weighted means (standard errors (SE)), and categorical variables as weighted percentages (SE). Differences in continuous variable weighted means were assessed using Student’s t-test, while differences in categorical variable weighted percentages were assessed using Cochran–Mantel–Haenszel Chi-square test. Weighted linear regression analyses were conducted to examine the relationships between UPFs as continuous variables or categorized into quartiles and PhenoAgeAccel. Model 1 without adjustments. Model 2 was additionally adjusted for age group, sex, race, Model 3 was additionally adjusted for PIR, education, physical activity, smoke status, drinks, hypertension, DM, CVD, HEI-2015, energy (kcal), and protein(g). Model 4 was additionally adjusted for BMI. Additionally, restricted cubic spline (RCS) analysis was conducted to explore the dose–response relationship between UPFs (as continuous variables) and PhenoAgeAccel after adjusting for all confounding variables. Subgroup and interaction analysis were also performed. Furthermore, the potential mediating role of BMI in the association between UPFs and PhenoAgeAccel was evaluated using the R package ‘mediation’.

Additionally, to address the lack of weighting in the mediation analysis and the potential influence of certain subpopulations, we conducted two sensitivity analyses. First, we performed an unweighted regression analysis. Second, we excluded participants who were pregnant or breasts feeding, had cancer, were aged over 80 years, or had abnormal or incomplete energy intake records (<500 or > 5,000 kcal/day for females; <500 or > 8,000 kcal/day for males). All statistical analyses were performed using R software (version 4.3.0).

## Results

### Baseline characteristics of study participants

The study analyzed 12,079 participants from the NHANES 2005–2010 cycles. Table 1 outlines the demographic and behavioral characteristics by aging group. Participants with accelerated aging were more likely to be older, have a higher percentage of daily intake from UPFs (%Gram), higher BMI, be male, non-Hispanic Black, have a lower PIR, lower education (high school or less), be active smokers, former drinkers, have a lower HEI-2015, and lower daily energy and protein intake. Additionally, they exhibited a higher prevalence of chronic diseases such as hypertension, diabetes, and CVD. Detailed demographic and behavioral characteristics are presented in Table 1.

**Table 1:** The baseline characteristics classified by aging groups in US adults, NHANES 2005–2010 (*n* = 12,079).

Variable	Total	Delayed (*n* = 9,306)	Accelerated (*n* = 2,773)	*p* value
**PhenoAgeAccel**	−4.27 (0.13)	−6.76 (0.08)	6.72 (0.20)	< 0.001
**Phenotypic age**	42.55 (0.42)	39.03 (0.39)	58.16 (0.56)	< 0.001
**Chronological age**	46.83 (0.36)	45.79 (0.37)	51.44 (0.54)	< 0.001
**Age groups**				< 0.001
< 40	36.56 (0.01)	38.74 (0.99)	26.90 (1.18)	
40–59	39.95 (0.02)	40.11 (0.72)	39.25 (1.46)	
≥ 60	23.49 (0.01)	21.15 (0.82)	33.85 (1.50)	
**UPFs (%Kcal)**	0.51 (0.00)	0.51 (0.00)	0.52 (0.01)	0.1
**UPFs (%Gram)**	0.35 (0.01)	0.34 (0.01)	0.37 (0.01)	< 0.001
**BMI**	28.69 (0.13)	27.81 (0.12)	32.59 (0.26)	< 0.001
**BMI groups**				< 0.001
Normal	31.52 (0.01)	34.79 (1.00)	17.05 (1.05)	
Overweight	33.45 (0.01)	35.00 (0.65)	26.61 (1.21)	
Obesity	35.03 (0.02)	30.21 (0.83)	56.34 (1.26)	
**Sex**				0.02
Male	49.09 (0.02)	48.39 (0.50)	52.17 (1.39)	
Female	50.91 (0.02)	51.61 (0.50)	47.83 (1.39)	
**Race/ethnicity**				< 0.001
Non-Hispanic White	72.98 (0.04)	74.31 (1.79)	67.10 (2.48)	
Non-Hispanic Black	10.20 (0.01)	8.68 (0.83)	16.88 (1.59)	
Mexican American	7.78 (0.01)	7.81 (0.90)	7.65 (1.16)	
Others	9.04 (0.01)	9.19 (0.84)	8.37 (0.98)	
**PIR**				< 0.001
Low	19.34 (0.01)	17.42 (0.85)	27.81 (1.29)	
Middle	34.97 (0.02)	34.12 (1.10)	38.73 (1.13)	
High	45.69 (0.02)	48.46 (1.41)	33.46 (1.58)	
**Education**				< 0.001
Middle school or lower	5.47 (0.00)	4.93 (0.39)	7.89 (0.68)	
High school	36.03 (0.02)	33.91 (1.19)	45.55 (1.75)	
College or more	58.44 (0.02)	61.16 (1.30)	46.57 (1.77)	
**Smoke**				< 0.001
Never	52.35 (0.02)	54.69 (0.98)	42.00 (1.40)	
Former	24.90 (0.01)	24.48 (0.77)	26.78 (1.28)	
Now	22.75 (0.01)	20.83 (0.65)	31.21 (1.33)	
**Drinks**				< 0.001
Former	15.95 (0.01)	14.23 (0.62)	23.53 (1.21)	
Mild	34.74 (0.02)	35.47 (0.94)	31.51 (0.97)	
Never	10.41 (0.01)	10.22 (0.69)	11.23 (0.76)	
Moderate	16.68 (0.01)	17.63 (0.67)	12.49 (0.87)	
Heavy	22.22 (0.01)	22.44 (0.77)	21.23 (0.77)	
**Physical activity**				< 0.001
Inactive	25.97 (0.01)	25.87 (0.95)	26.44 (1.41)	
Moderate	13.51 (0.01)	14.09 (0.57)	10.94 (0.86)	
Active	41.01 (0.02)	42.68 (1.19)	33.63 (1.40)	
Others	19.51 (0.01)	17.36 (0.65)	28.99 (1.14)	
**Hypertension**	36.19 (0.02)	32.45 (0.84)	52.74 (1.56)	< 0.001
**DM**	12.18 (0.01)	6.96 (0.34)	35.23 (1.43)	< 0.001
**CVD**	6.04 (0.00)	4.36 (0.29)	13.49 (0.67)	< 0.001
**HEI-2015**	50.70 (0.32)	51.24 (0.34)	48.32 (0.38)	< 0.001
**Energy (kcal)**	2177.66 (15.69)	2194.51 (14.60)	2103.13 (38.79)	0.02
**Protein (g)**	84.47 (0.69)	85.12 (0.68)	81.59 (1.66)	0.04

Weighted mean +/− Se and Student’s *t*-test for continuous variables. Weighted %, mean (95% CI), and Cochran–Mantel–Haenszel Chi-square test for categorical variables.

### Associations between UPFs and PhenoAgeAccel

Table 2 presents the associations between UPFs intake and PhenoAgeAccel. After adjusting for all covariates, no significant association was found between UPF intake (%Kcal) and PhenoAgeAccel. However, a positive association was observed between UPFs intake (%Gram) and PhenoAgeAccel (*β* = 0.98, 95% CI: 0.17, 1.78; *p* = 0.02), with participants in the highest quartile of UPFs intake (%Gram) showing an increase of 0.6 years (95% CI: 0.15, 1.05; p for trend = 0.039) compared to those in the lowest quartile.

**Table 2:** Association between UPFs (%Kcal and %Gram) and PhenoAgeAccel in US adults in NHANES 2005–2010 (*n* = 12,079).

	UPFs (%Kcal)		UPFs (%Gram)	
Character	95% CI	*P* value	95% CI	*P* value
**Continuous**				
**Model 1**	0.82 (−0.17,1.81)	0.1	2.68 (1.88,3.47)	<0.001
**Model 2**	1.4 (0.42, 2.38)	0.01	2.85 (2.06, 3.65)	<0.001
**Model 3**	0.72 (−0.33, 1.76)	0.17	1.41 (0.57, 2.24)	0.002
**Model 4**	0.38 (−0.61, 1.37)	0.44	0.98 (0.17, 1.78)	0.02
**Quantiles**				
**Model 1**				
Q1	Ref	Ref	Ref	Ref
Q2	−0.6 (−1.06, −0.15)	0.01	0.49 (0.12, 0.86)	0.01
Q3	−0.02 (−0.53, 0.49)	0.94	0.42 (−0.05,0.90)	0.08
Q4	0.22 (−0.31, 0.75)	0.41	1.49 (1.02, 1.96)	<0.001
*P* for trends	0.116		0.001	
**Model 2**				
Q1	Ref	Ref	Ref	Ref
Q2	−0.4 (−0.85, 0.05)	0.08	0.41 (0.02, 0.80)	0.04
Q3	0.16 (−0.39, 0.71)	0.56	0.4 (−0.08, 0.88)	0.1
Q4	0.56 (0.06, 1.06)	0.03	1.52 (1.04, 2.00)	<0.001
*P* for trends	0.005		0.001	
**Model 3**				
Q1	Ref	Ref	Ref	Ref
Q2	−0.23 (−0.63, 0.16)	0.23	0.43 (0.09, 0.77)	0.02
Q3	0.15 (−0.40, 0.70)	0.58	0.14 (−0.27, 0.55)	0.49
Q4	0.22 (−0.30, 0.73)	0.4	0.83 (0.36, 1.29)	0.001
*P* for trends	0.221		0.004	
**Model 4**				
Q1	Ref	Ref	Ref	Ref
Q2	−0.24 (−0.63, 0.14)	0.2	0.38 (0.06, 0.69)	0.02
Q3	0.11 (−0.44, 0.66)	0.68	0.06 (−0.32, 0.43)	0.75
Q4	0.06 (−0.44, 0.57)	0.79	0.6 (0.15, 1.05)	0.01
*P* for trends	0.51		0.039	

Model 1 without adjustments. Model 2 was additionally adjusted for age group, sex, race, Model 3 was additionally adjusted for PIR, education, physical activity, smoke status, drinks, hypertension, DM, CVD, HEI-2015, energy (kcal), and protein(g). Model 4 was additionally adjusted for BMI.

### Dose–response relationship between UPFs and PhenoAgeAccel

**Figure 2:**
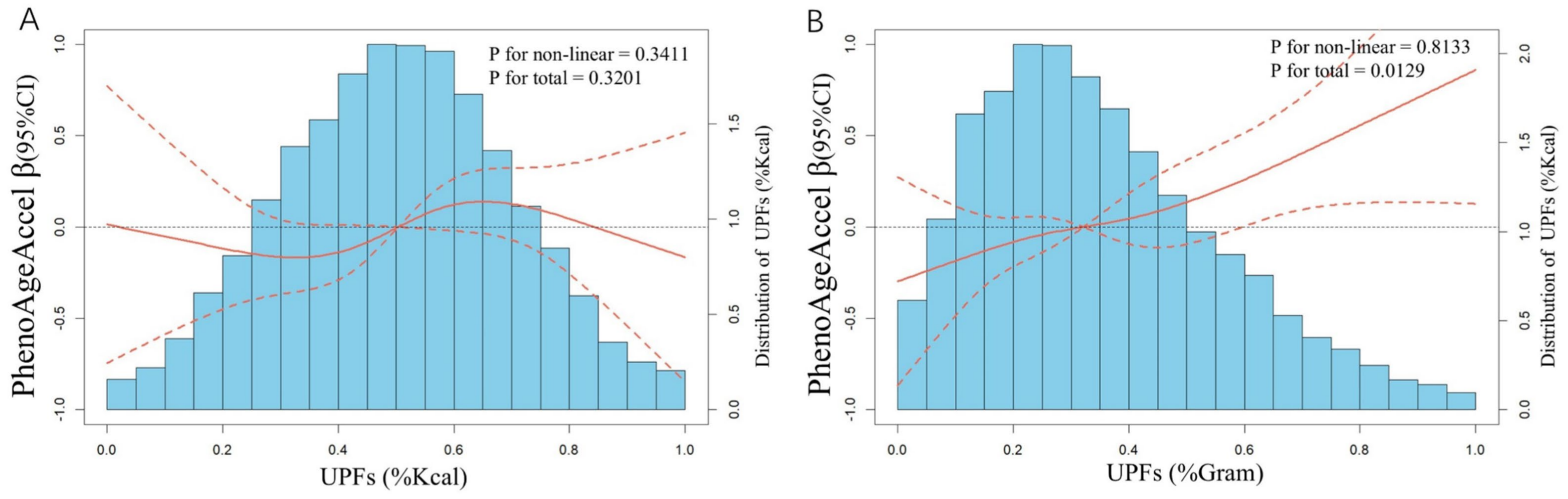
Distributions of frequency of UPFs intake (%Kcal and %Gram) and dose–response relationship between UPFs intake (**A**: %Kcal and **B**: %Gram) and PhenoAgeAccel in US adults (*n* = 12,079), NHANES 2005 to 2010. Values represent difference in predicted response in reference to a UPFs intake (%Kcal and %Gram) of mean. Red solid lines and Red dotted line represent restricted cubic spline models and 95%CI, respectively. Multivariable linear regression models were used to estimate the fully adjusted beta coefficient in PhenoAgeAccel and corresponding 95% CI. Models were adjusted by age group, sex, race/ethnicity, BMI, PIR, education, physical activity, smoke status, drinks, hypertension, DM, CVD, HEI-2015, energy (kcal), and protein(g).

Figure 2 illustrates the dose–response relationships between UPFs intake and PhenoAgeAccel using restricted cubic splines (RCS). No significant association was found between UPFs intake (%Kcal) and PhenoAgeAccel (Figure 2A). However, a linear positive correlation was identified between UPFs intake (%Gram) and PhenoAgeAccel (p for non-linearity = 0.8133; p for overall effect <0.0129) (Figure 2B).

### Subgroup and interaction analyses

Supplementary Table S1 presents the results of stratified analyses and interaction tests between UPFs intake (%Kcal and %Gram) and PhenoAgeAccel. The association between UPFs intake (%Kcal) and PhenoAgeAccel did not significantly vary across most subgroups, except for race/ethnicity. Notably, stronger positive associations were found between UPFs intake (%Gram) and PhenoAgeAccel within specific race/ethnicity groups and among former drinkers, with significant interactions (p for interaction = 0.002 and 0.03, respectively).

### Mediation analysis

**Figure 3:**
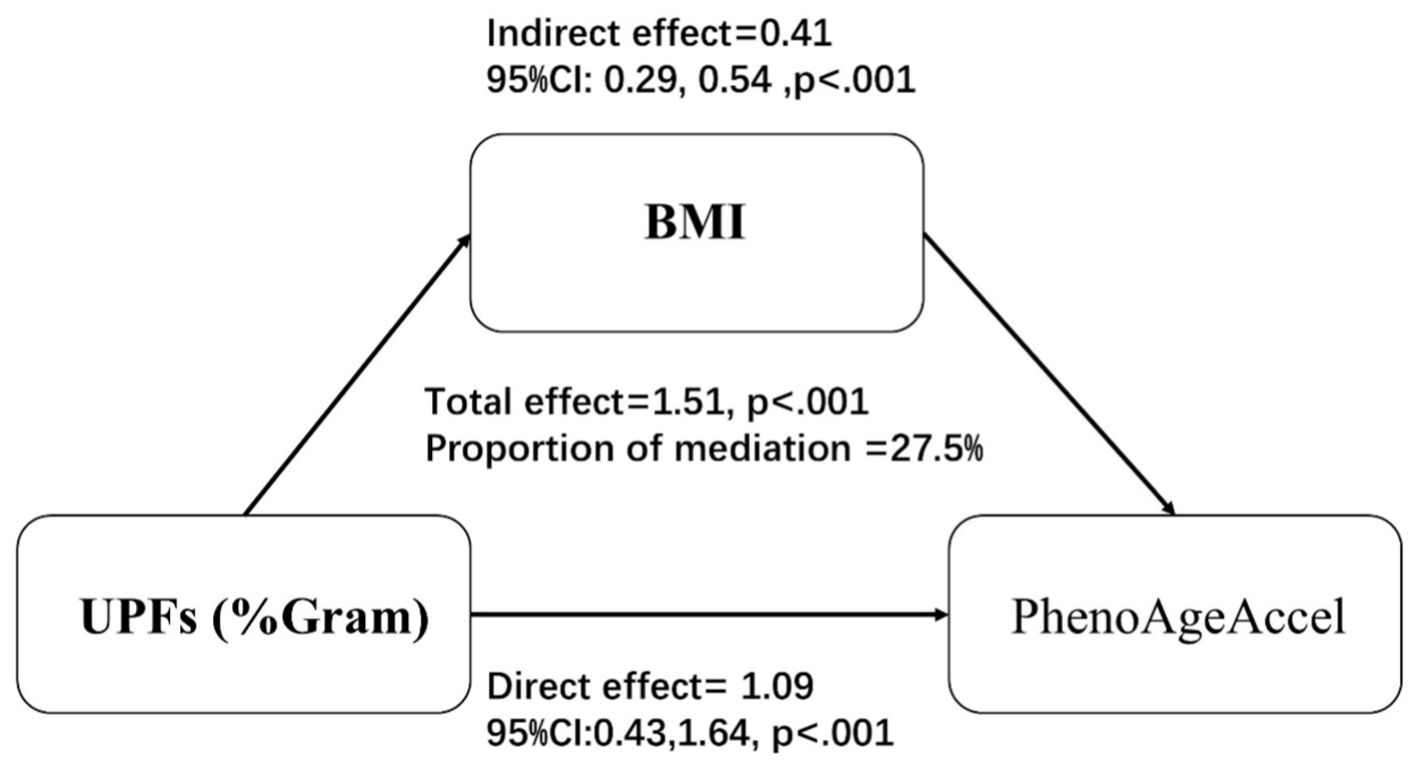
Mediation effects of BMI on the association between UPFs intake (%Gram) and PhenoAgeAccel. Models were Adjusted for age group, sex, race/ethnicity, BMI, PIR, education, physical activity, smoke status, drinks, hypertension, DM, CVD, HEI-2015, energy (kcal), and protein(g).

Mediation analysis, illustrated in Figure 3, was conducted to assess the mediating effect of BMI on the relationship between UPFs intake (%Gram) and PhenoAgeAccel. The analysis revealed that BMI significantly mediated 27.5% of the association between UPF intake (%Gram) and PhenoAgeAccel.

### Sensitivity analysis

Sensitivity analyses confirmed the robustness of the findings. No significant association was observed between UPFs intake (%Kcal) and PhenoAgeAccel in both unweighted analyses (Supplementary Table S2) and after excluding certain subpopulations with potentially significant influence (Supplementary Table S3). In contrast, UPFs intake (%Gram) consistently showed a positive association with PhenoAgeAccel, with effect estimates of *β* = 1.01 (95% CI: 0.32, 1.70; *p* = 0.02) and *β* = 0.86 (95% CI: 0.04, 1.67; *p* = 0.04) in these sensitivity analyses. Additionally, participants in the highest quartile of UPFs intake (%Gram) exhibited an increase in PhenoAgeAccel by 0.54 years (95% CI: 0.15, 0.94; *p* for trend = 0.013) and 0.63 years (95% CI: 0.21, 1.05; p for trend = 0.016) compared to those in the lowest quartile, consistent with the primary findings.

## Discussion

Our study presents novel insights into the differential associations of UPFs intake measured by grams (%Gram) and calories (%Kcal) with PhenoAgeAccel. The findings indicate that while UPFs intake (%Gram) is positively associated with PhenoAgeAccel, no significant association was observed for UPFs intake (%Kcal). This finding suggests that the quantity of UPFs consumed, rather than their caloric contribution, plays a more critical role in influencing phenotypic aging.

The aging process is influenced by both genetic and epigenetic factors. Twelve key features of biological aging have been identified, including genomic instability, telomere attrition, epigenetic alterations, loss of proteostasis, disabled macroautophagy, deregulated nutrient-sensing, mitochondrial dysfunction, cellular senescence, stem cell exhaustion, altered intercellular communication, chronic inflammation, and dysbiosis (19). These characteristics offer a comprehensive framework for understanding the multifactorial nature of biological aging, which is critical when examining the impacts of diet on aging processes. We can assess biological aging through various methods, among which phenotypic age derived from serum biomarkers and clinical features are widely used in clinical practice. Phenotypic age is based on external physiological characteristics and uses key health indicators and physiological parameters to assess an individual’s overall health status, reflecting the degree of aging in appearance and function, which provides a high correlation with actual age and demonstrates strong predictive power for mortality, age-related diseases, comorbidity, and physical decline (20).

Currently, UPFs are increasingly infiltrating traditional diets, especially in high- and middle-income countries (21) and also in low-income countries (22). Numerous observational studies have linked high UPFs consumption with a range of health risk factors in adults, which are linked to higher mortality (23). These health risks are often closely associated with the aging process, making diet a crucial modifiable factor influencing aging. Previous research has shown that various dietary components and patterns can affect aging by influencing inflammation and oxidation across multiple organs and tissues (24–26).UPFs may exacerbate oxidative stress and chronic inflammation, which are well-established drivers of chronic diseases and accelerate the aging process (27, 28). Moreover, UPFs might also influence aging by disrupting the balance of the gut microbiome, further exacerbating inflammatory responses (29, 30). The positive association between UPFs intake (%Gram) and PhenoAgeAccel can be attributed to the higher physical volume of UPFs consumed, which likely has more direct impacts on metabolic health and inflammatory processes. The gram measurement reflects the physical volume of food consumed, which can displace healthier, nutrient-dense options, potentially leading to nutrient deficiencies and increased exposure to harmful substances in UPFs. This displacement can degrade dietary quality and increase exposure to harmful additives and preservatives in UPFs, thereby accelerating aging.

While UPFs (%Gram) intake was positively associated with PhenoAgeAccel, no such association was observed for UPFs (%Kcal), suggesting that caloric content alone may not fully capture the detrimental effects of UPFs. This discrepancy may arise because measuring UPFs by grams reflects the physical quantity consumed, which more accurately represents the intake of additives and preservatives that may accelerate aging by affecting cellular function (31) and metabolic pathways (32). In contrast, measuring UPFs by calories might dilute these effects due to variations in energy density among different UPFs. Larger portions of UPFs, measured by weight, could also contribute to overeating and an increased metabolic burden, as their intense flavors can override natural satiety mechanisms (33, 34). Furthermore, our subgroup analysis revealed that the negative impact of % daily grams intake from UPFs on biological age varied across different racial and drinks groups, potentially due to psychosocial factors, dietary preferences, or lifestyle choices specific to these populations (35).

Our mediation analysis further supports the role of BMI as a significant mediator in the relationship between UPFs (%Gram) and PhenoAgeAccel. The analysis revealed that BMI accounted for a substantial portion of the association, indicating that the impact of UPFs on phenotypic aging may be largely driven by their contribution to obesity. This finding is consistent with previous research on dietary patterns, such as the Dietary Inflammatory Index, Healthy Eating Index-2020, Alternative Healthy Eating Index-2010, and Composite Dietary Antioxidant Index, and their associations with aging acceleration (24). Obesity, characterized by chronic low-grade inflammation and increased oxidative stress, is well-documented to accelerate aging processes (36). The fact that BMI partially mediated the relationship underscores the importance of body weight management in mitigating the adverse effects of UPFs consumption on aging.

The strength of this study lies in its novel identification of the complex association between UPFs consumption and aging acceleration, particularly highlighting the significant mediating role of BMI. Additionally, the study’s large sample size enhances statistical power, and the cross-sectional design provides a comprehensive snapshot of the associations between UPFs intake and aging acceleration. However, this study also has several limitations. As a cross-sectional study, it cannot establish causal relationships. Moreover, the reliance on self-reported dietary intake data introduces the possibility of recall bias, which could affect the true association between UPFs and aging acceleration. Furthermore, although the NOVA classification system was used to categorize UPFs, its application to NHANES data may have led to some misclassification, which could impact the accuracy of UPFs categorization.

## Conclusion

In summary, our findings suggest that the total volume of UPFs consumed, as indicated by %Gram, is more relevant to PhenoAgeAccel than their caloric contribution. Additionally, BMI plays a significant mediating role in this relationship, highlighting the importance of controlling obesity as a potential strategy to mitigate the aging effects of unhealthy diets. These findings highlight the importance of considering not just the caloric intake but the physical volume of UPFs in dietary recommendations aimed at mitigating aging-related health risks. Future longitudinal studies are needed to establish causal relationships and to explore the underlying mechanisms in more detail.

## Data Availability

Publicly available datasets were analyzed in this study. This data can be found at: https://wwwn.cdc.gov/nchs/nhanes/Default.aspx.
