# Supplementary material for: Association of ultra-processed foods with phenotypic age acceleration in US adults: a mediation analysis of body mass index in the NHANES
Source: Front Nutr. 2025 Mar 24;12:1485456. doi: 10.3389/fnut.2025.1485456 (PMC11973088; doi:10.3389/fnut.2025.1485456)
Supplement: Supplementary file 1 [file Table_1.DOCX]

Table S1. Estimated changes of phenotypic age with UPFs intake (%Kcal AND %Gram) stratified by subgroups.

|  | **UPFs %Kcal）** |  |  | **UPFs %Gram）** |  |
| --- | --- | --- | --- | --- | --- |
| **character** | **95% CI** | **P^*^** |  | **95% CI** | **p^*^** |
| **Age group** |  | 0.83 |  |  | 0.37 |
| <40 | 0.91(-0.44, 2.25) |  |  | 0.67(-0.45, 1.80) |  |
| ≥60 | -0.25(-2.37, 1.88) |  |  | 0.2(-1.67, 2.07) |  |
| 40-59 | -0.27(-1.77, 1.22) |  |  | 1.43(0.14, 2.73) |  |
| **Sex** |  | 0.14 |  |  | 0.78 |
| Male | -0.16(-1.76, 1.45) |  |  | 1.08(0.01, 2.14) |  |
| Female | 0.84(-0.40, 2.07) |  |  | 0.69(-0.58, 1.95) |  |
| **Race/ethnicity** |  | < 0.001 |  |  | 0.02 |
| Non-Hispanic White | -0.17(-1.41, 1.08) |  |  | 0.61(-0.36, 1.58) |  |
| Non-Hispanic Black | -1.03(-3.03, 0.97) |  |  | 1.68(-0.17, 3.53) |  |
| Mexican American | 0.4(-2.28, 3.08) |  |  | -0.04(-2.04, 1.95) |  |
| others | 4.4(2.02,6.79) |  |  | 3.72(1.03,6.41) |  |
| **PIR** |  | 0.38 |  |  | 0.43 |
| low | 0.78(-0.88, 2.43) |  |  | 1.37(0.21, 2.53) |  |
| middle | 0.43(-1.17, 2.02) |  |  | 1(-0.05, 2.06) |  |
| high | 0.18(-1.04, 1.40) |  |  | 0.83(-0.43, 2.08) |  |
| **Education** |  | 0.71 |  |  | 0.79 |
| Middle school or lower | 1.51(-2.42, 5.44) |  |  | 1.34(-2.60, 5.29) |  |
| High school | 0.22(-1.13, 1.57) |  |  | 0.86(-0.32, 2.05) |  |
| College or more | 0.32(-0.94, 1.59) |  |  | 0.92(-0.15, 1.99) |  |
| **Physical activity** |  | 0.36 |  |  | 0.13 |
| inactive | 1.09(-0.52, 2.70) |  |  | 1.93(0.57, 3.28) |  |
| moderate | 0.19(-2.34, 2.72) |  |  | 1.69(-1.26, 4.64) |  |
| active | 0.34(-1.04, 1.73) |  |  | 0.95(-0.14, 2.03) |  |
| others | -0.54(-2.60, 1.51) |  |  | -0.34(-2.18, 1.50) |  |
| **Smoke** |  | 0.73 |  | 2(0.93, 3.08) |  |
| never | 0.74(-0.49, 1.97) |  |  | -0.15(-1.67, 1.37) |  |
| former | -0.25(-2.28, 1.79) |  |  | 0.37(-1.10, 1.84) |  |
| now | 0.33(-1.66, 2.32) |  |  |  |  |
| **Drinks** |  | 0.42 |  |  | 0.03 |
| former | 1.31(-1.18, 3.81) |  |  | 1.16(-0.60, 2.92) |  |
| mild | 0.65(-0.95, 2.25) |  |  | 2.16(0.74, 3.59) |  |
| never | 1.48(-0.95, 3.92) |  |  | 2.15(-0.28, 4.59) |  |
| moderate | 0.45(-1.52, 2.42) |  |  | 0.43(-1.21, 2.07) |  |
| heavy | -0.81(-2.22,0.60) |  |  | -0.56(-2.02,0.89) |  |
| **BMI group** |  | 0.59 |  |  | 0.79 |
| normal | 1.02(-0.38, 2.43) |  |  | 1.04(-0.32, 2.41) |  |
| overweight | 0.03(-1.77, 1.83) |  |  | 1.49( 0.14, 2.83) |  |
| obesity | 0.19(-1.37, 1.76) |  |  | 0.59(-0.66, 1.84) |  |

Models were adjusted by age group, sex, race/ethnicity, BMI, PIR, education, physical activity, smoke, drinks, hypertension, DM, CVD, HEI-2015, energy (kcal), and protein(g). The subgroup variable was not included in same subgroup analysis.

Ref: reference.

P*：P for interaction.

Table S2. Unweighted Association between UPFs intake (%Kcal and %Gram) and PhenoAgeAccel in US adults in NHANES 2005–2010 (n = 12079).

|  | **UPFs %Kcal）** | |  | **UPFs %Gram）** | |
| --- | --- | --- | --- | --- | --- |
| **character** | **95% CI** | **P value** |  | **95% CI** | **P value** |
| **Continuous** |  |  |  |  |  |
| **model 1** | 0.34(-0.41,1.09) | 0.37 |  | 2.11(1.40,2.83) | <.001 |
| **model 2** | 1.13(0.36, 1.89) | 0.004 |  | 2.54(1.82, 3.27) | <.001 |
| **model 3** | 0.64(-0.11, 1.38) | 0.09 |  | 1.42(0.72, 2.12) | <.001 |
| **model 4** | 0.17(-0.56, 0.90) | 0.65 |  | 1.01(0.32, 1.70) | 0.004 |
| **Quantiles** |  |  |  |  |  |
| **model 1** |  |  |  |  |  |
| Q1 | ref | ref |  | ref | ref |
| Q2 | -0.56(-0.98, -0.14) | 0.01 |  | 0.22(-0.20,0.64) | 0.31 |
| Q3 | -0.25(-0.67, 0.17) | 0.24 |  | 0.2(-0.21,0.62) | 0.34 |
| Q4 | 0.01(-0.41, 0.43) | 0.97 |  | 1.16(0.74,1.58) | <.001 |
| P for trends | 0.684 |  |  | <.001 |  |
| **model 2** |  |  |  |  |  |
| Q1 | ref | ref |  | ref | ref |
| Q2 | -0.4(-0.82, 0.01) | 0.06 |  | 0.14(-0.27, 0.55) | 0.5 |
| Q3 | -0.07(-0.49, 0.35) | 0.74 |  | 0.17(-0.24, 0.58) | 0.42 |
| Q4 | 0.42(-0.01, 0.85) | 0.05 |  | 1.3(0.88, 1.72) | <.001 |
| P for trends | 0.025 |  |  | <.001 |  |
| **model 3** |  |  |  |  |  |
| Q1 |  |  |  | ref | ref |
| Q2 | ref | ref |  | 0.24(-0.14, 0.61) | 0.22 |
| Q3 | -0.26(-0.64, 0.12) | 0.18 |  | -0.01(-0.39, 0.38) | 0.97 |
| Q4 | -0.02(-0.42, 0.37) | 0.9 |  | 0.77(0.37, 1.18) | <0.001 |
| P for trends | 0.13(-0.27, 0.54) | 0.52 |  | <0.001 |  |
| model 4 | 0.361 |  |  |  |  |
| Q1 |  |  |  |  |  |
| Q2 | ref | ref |  | ref | ref |
| Q3 | -0.35(-0.72, 0.02) | 0.07 |  | 0.14(-0.23, 0.51) | 0.47 |
| Q4 | -0.14(-0.52, 0.25) | 0.49 |  | -0.1(-0.47, 0.28) | 0.61 |
| P for trends | -0.11(-0.51, 0.29) | 0.6 |  | 0.54(0.15, 0.94) | 0.01 |
|  | 0.823 |  |  | 0.013 |  |

Model 1 without adjustments. Model 2 was additionally adjusted for age group, sex, race, Model 3 was additionally adjusted for PIR, education, physical activity, smoke status, drinks, hypertension, DM, CVD, HEI-2015, energy (kcal), and protein(g). Model 4 was additionally adjusted for BMI.

Table S3. Association between UPFs (%Kcal and %Gram) and PhenoAgeAccel in selected US adults in NHANES 2005–2010 (n = 10242).

|  | **UPFs %Kcal）** | |  | **UPFs %Gram）** | |
| --- | --- | --- | --- | --- | --- |
| **character** | **95% CI** | **P value** |  | **95% CI** | **P value** |
| **Continuous** |  |  |  |  |  |
| **model 1** | 0.91(-0.06,1.88) | 0.07 |  | 2.69(1.87,3.51) | <.001 |
| **model 2** | 1.35(0.43, 2.26) | 0.005 |  | 2.72(1.88, 3.56) | <.001 |
| **model 3** | 0.77(-0.18, 1.72) | 0.11 |  | 1.36(0.51, 2.21) | 0.003 |
| **model 4** | 0.39(-0.53, 1.30) | 0.39 |  | 0.86(0.04, 1.67) | 0.04 |
| **Quantiles** |  |  |  |  |  |
| **model 1** |  |  |  |  |  |
| Q1 | ref | ref |  | ref | ref |
| Q2 | -0.86(-1.23, -0.48) | <.001 |  | 0.43(0.01,0.85) | 0.05 |
| Q3 | -0.09(-0.56, 0.38) | 0.7 |  | 0.46(-0.02,0.95) | 0.06 |
| Q4 | 0.25(-0.30, 0.79) | 0.37 |  | 1.46(0.96,1.97) | <.001 |
| P for trends | 0.073 |  |  | <.001 |  |
| **model 2** |  |  |  |  |  |
| Q1 | ref | ref |  | ref | ref |
| Q2 | -0.67(-1.03, -0.31) | <0.001 |  | 0.35(-0.08, 0.77) | 0.1 |
| Q3 | 0.08(-0.41, 0.56) | 0.75 |  | 0.45(-0.03, 0.93) | 0.07 |
| Q4 | 0.51(0.01, 1.02) | 0.05 |  | 1.42(0.90, 1.94) | <.001 |
| P for trends | 0.005 |  |  | <.001 |  |
| **model 3** |  |  |  |  |  |
| Q1 | ref | ref |  | ref | ref |
| Q2 | -0.35(-0.70, 0.00) | 0.05 |  | 0.36(0.00, 0.73) | 0.05 |
| Q3 | 0.11(-0.41, 0.63) | 0.68 |  | 0.12(-0.32, 0.56) | 0.57 |
| Q4 | 0.24(-0.27, 0.74) | 0.35 |  | 0.74(0.25, 1.23) | 0.005 |
| P for trends | 0.154 |  |  | 0.01 |  |
| model 4 |  |  |  |  |  |
| Q1 | ref | ref |  | ref | ref |
| Q2 | -0.33(-0.69, 0.03) | 0.07 |  | 0.37(0.05, 0.68) | 0.02 |
| Q3 | 0.04(-0.47, 0.56) | 0.86 |  | 0.11(-0.27, 0.50) | 0.55 |
| Q4 | 0.09(-0.42, 0.59) | 0.73 |  | 0.63(0.21, 1.05) | 0.005 |
| P for trends | 0.433 |  |  | 0.016 |  |

Model 1 without adjustments. Model 2 was additionally adjusted for age group, sex, race, Model 3 was additionally adjusted for PIR, education, physical activity, smoke status, drinks, hypertension, DM, CVD, HEI-2015, energy (kcal), and protein(g). Model 4 was additionally adjusted for BMI.
